# Effect of Fc core fucosylation and light chain isotype on IgG1 flexibility

**DOI:** 10.1038/s42003-023-04622-7

**Published:** 2023-03-03

**Authors:** Simona Saporiti, Tommaso Laurenzi, Uliano Guerrini, Crescenzo Coppa, Wolf Palinsky, Giulia Benigno, Luca Palazzolo, Omar Ben Mariem, Linda Montavoci, Mara Rossi, Fabio Centola, Ivano Eberini

**Affiliations:** 1 Dipartimento di Scienze Farmacologiche e Biomolecolari, Università degli Studi di Milano, Via Balzaretti, 9, 20133 Milan, Italy; 2 Dipartimento di Scienze Farmaceutiche, Università degli Studi di Milano, Sezione di Chimica Generale e Organica “A. Marchesini”, Via Venezian, 21, 20133, Milano, Italy; 3 Biotech Development Programme, Merck Biopharma, Aubonne, Switzerland; 4 Global Analytical Pharmaceutical Science and Innovation, Merck Serono S.p.A., Rome, Italy; 5 Dipartimento di Scienze Farmacologiche e Biomolecolari & DSRC, Università degli Studi di Milano, Via Balzaretti, 9, 20133 Milan, Italy

**Keywords:** Protein function predictions, Protein structure predictions

## Abstract

N-glycosylation plays a key role in modulating the bioactivity of monoclonal antibodies (mAbs), as well as the light chain (LC) isotype can influence their physicochemical properties. However, investigating the impact of such features on mAbs conformational behavior is a big challenge, due to the very high flexibility of these biomolecules. In this work we investigate, by accelerated molecular dynamics (aMD), the conformational behavior of two commercial immunoglobulins G1 (IgG1), representative of κ and λ LCs antibodies, in both their fucosylated and afucosylated forms. Our results show, through the identification of a stable conformation, how the combination of fucosylation and LC isotype modulates the hinge behavior, the Fc conformation and the position of the glycan chains, all factors potentially affecting the binding to the FcγRs. This work also represents a technological enhancement in the conformational exploration of mAbs, making aMD a suitable approach to clarify experimental results.

## Abstract (web summary)

Classical and accelerated molecular dynamics simulations reveal how core fucosylation in the IgG1 Fc glycan and the light chain isotype modulate the conformational behavior of the hinge and Fab domains and dynamics of IgG1 antibodies.

## Introduction

Most of the clinically available monoclonal antibodies (mAbs) are immunoglobulin G1 (IgG1)^1^, because of their higher stability and potent effector functions with respect to other IgG subclasses. mAbs are composed of three domains: two fragment antigen binding domains (Fab) and one crystallizable fragment (Fc), including heavy and light chains (HC and LC, respectively), both containing variable and constant regions. The variable domains are responsible for the adaptive immune response, or, in the case of commercial mAbs, for selectively binding a target antigen. Antibodies can present two different isotypes of LC, namely *κ* and *λ*^2^. The ratio of antibodies containing *κ* or *λ* LCs varies considerably among species^3^ and, considering the ~ 100 approved therapeutic mAbs, only few contain a *λ* LC^4^. Few studies are published on the functional and structural comparison between these two isotypes, suggesting differences in Fab domains cooperativity and flexibility^5^, and in the structural properties of complementarity determining regions (CDRs)^6^. The ability of IgG1s to activate the immune system by the interaction of the Fc with specific Fcγ receptors (FcγR) is considered a key aspect also regulated by N-glycosylation on the conserved Asn297 in the Fc^7,8^. Alteration in the length, composition, and charge of the glycans can impact the structural integrity and conformation of the Fc domain, thus changing the binding affinity to FcγRs and influencing the immune response^9,10^. In particular, core fucosylation can affect the antibody-dependent cell cytotoxicity (ADCC), since it decreases the binding affinity of the IgG1 to the FcγRIIIa (a low affinity activated receptor)^1,11–17^.

Despite these observations, the structural role of LC differences in modulating the functional behavior of these biomolecules, both in terms of antigen recognition and effector function activation, has never been investigated. Some studies^18,19^, have proposed hypotheses to explain the fucosylation effect on the effector functions, but focusing only on the Fc without considering the role of the hinge and Fab domains. In our previous work^20^, we proposed that the presence of fucose can modulate the conformational behavior of the whole mAb inducing a preference for a T-shaped conformation, in principle less suitable for receptor binding. In agreement with our previous results, Spiteri et al. have demonstrated how glycans can introduce structural constraints, by a comparison of glycosylated and aglycosylated IgG1. This work shows that the removal of glycans affects the Fab-Fc separation, by modulating the flexibility of the protein, letting the antibody to explore a different conformational space, and impacting the binding to the FcγRs^21^. In this work, we investigate the role of the fucose and of the two LC isotypes in the structural behavior of IgG1s, using an innovative in silico approach for mAbs, based on a combination of classical and accelerated molecular dynamics simulations (cMD and aMD, respectively). A comparison between the afucosylated (G0) and the fucosylated (G0F) form of adalimumab and avelumab, two commercial IgG1s that are good models of κ and λ LCs antibodies, was carried out, suggesting a key role of *λ* LCs in modulating the dynamics of IgG1. To the best of our knowledge, the combination of standard and enhanced sampling MD methods has never been used in the context of mAbs conformational behavior. Accordingly, our results can pave the way to new future perspectives (experimental and computational) in the investigation of the antibody flexibility.

## Results

### Analysis of cMD trajectories

**Fig. 1:**
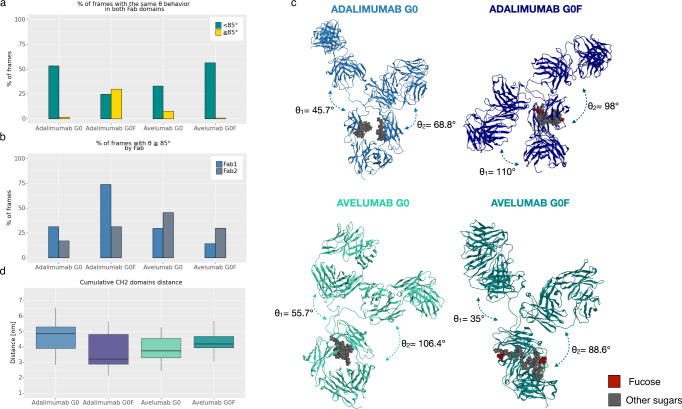
Analysis of cMD trajectories. **a** Bar plot showing the percentage of frames in which θ angles of both Fab domains present values ≥ or <85° simultaneously. **b** Bar plot showing the percentage of frames in which θ angle of each Fab domain is ≥85°. **c** Medoids of the most populated cluster identified for each mAb with the corresponding θ values. **d** Box plots of CH2 domains distance distribution for each mAb. Plots shown in (**a**, **b** and **d**) were generated by merging three replicas for each system with *n* = 21,000 frames for each condition.

Chimeric 3D models were built for adalimumab and avelumab, chosen as good models of κ and λ isotypes, as described in Materials and Methods section. The selection of these two commercial mAbs as case study was performed according to a sequence alignment shown in Supplementary material (Supplementary Fig. 1). Two afucosylated (adalimumab G0, avelumab G0) and two fucosylated (adalimumab G0F, avelumab G0F) models were obtained and simulated via cMD for a total of 3 µs (three 1 µs long replicas). A schematic representation of glycans used in this study is reported in Supplementary Fig. 2, according to the Symbol Nomenclature For Glycans (SNFG) scheme^22^. In these systems, the presence of the hinge region, a flexible linker that connects all the domains leaving at least four degrees of freedom (θ and ϕ for each Fab), prevents the convergence of root mean square deviation (RMSD) and RMS fluctuation (RMSF), that become poorly informative. In Supplementary Figs. 3–5 the RMSD, RMSF and Rg plots computed for C-alpha atoms are reported, respectively, showing that all the systems go out from the high fluctuation state of geometric parameters after 300 ns of cMD, even if none of them reaches a well-defined plateau state of these descriptors. Accordingly, the analysis was focused on the variation of θ angle in the last 7000 frames. This angle, as already shown^20^, represents the propensity of Fab domains to come close to the Fc, allowing the classification of Y- (θ < 85°) or T-shaped (θ ≥ 85°) conformations. Figure 1a shows a clear tendency of G0F adalimumab to stay in a T-shaped conformation, since for the 30% of time both Fab present θ ≥ 85°. This trend is inverted in G0 adalimumab that for up to 50% of time tends to assume a Y-shaped conformation. On the contrary, avelumab shows no significant differences between the G0 and G0F species, but both seem to prefer a Y-shaped orientation. Moreover, all the studied biopolymers present an asymmetric behavior, with a different distribution of the θ values assumed by single Fab domains along the trajectory (Fig. 1b). These conformational features were pointed out by a cluster analysis showing a clear Y-shaped conformation for G0 adalimumab, G0 and G0F avelumab, and a T-shaped conformation for G0F adalimumab (Fig. 1c).

According to the evidence by which the presence of an open or closed Fc conformation can be associated to a higher or lower FcγR binding affinity^23^, an analysis of the distance between CH2 domains is reported. The results (Fig. 1d) show that adalimumab G0F has a lower distance in terms of median value (3.1 nm) and therefore a closed Fc domain than adalimumab G0 (4.8 nm), as reported in our previous work^20^. Avelumab species show instead very similar distributions, with median values of 3.7 nm (G0) and 4.1 nm (G0F), comparable to adalimumab G0, supporting the hypothesis of very similar behaviors of avelumab forms. However, as shown in the next paragraphs, the cMD was not efficient enough to explore the conformational space of the λ LC IgG1s.

### Analysis of aMD trajectories

#### Analysis of θ angles

To further explore and characterize the conformational behavior of all the investigated species, 1 μs long aMD simulations were carried out. With this method a bias potential is added to the system, perturbing both the potential energy and the dihedral angles, and accelerating the conformational changes. This method allows the exploration of a wider region of the free energy surface (FES) of the molecule compared to a cMD, thus allowing also to sample conformational states not observed with the cMD simulations.

**Fig. 2:**
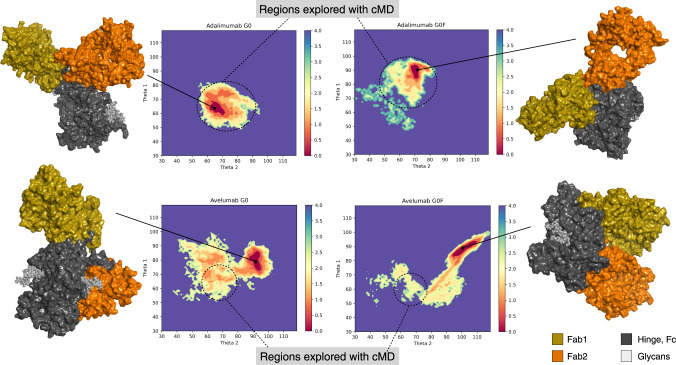
Free energy profile along Theta1 and Theta2. The free energy profile (after reweighting) of the four antibodies along Theta1 and Theta2 angles with the molecular surface of medoid structures isolated from the minimum energy region (in dark red) by cluster analysis. The color bar represents the PMF value in kcal/mol. Black dashed circles show the conformational space explored with cMD simulations, that for avelumab is limited with respect to that sampled via the aMD.

The free energy profile along Theta1 (θ angle between Fab1 and Fc) and Theta2 (θ angle between Fab2 and Fc) was computed to obtain the average force of all possible configurations of each system (Fig. 2). This analysis allowed the identification of a region of the FES with a minimum value of potential of mean force (PMF < 0.5 kcal/mol). The results on G0 and G0F adalimumab confirm the behavior observed with cMD, with a preferred Y-shaped conformation for G0 adalimumab (θ ≈ 70° for both Fab), and T-shaped conformation for G0F adalimumab (θ > 90° for both Fab), as shown in Fig. 2. On the opposite, both G0 and G0F avelumab are prone to reach a T-shaped conformation, with θ > 90° in at least one Fab in G0 avelumab, and in both Fab domains in G0F avelumab. According to this analysis, the role of fucose in promoting the T-shaped conformation is confirmed for both isotypes. On the other hand, a putative role of the *λ* LC in promoting a T-shaped conformation, even in the absence of fucose, is figured out. Moreover, especially for avelumab, these results clearly spotlight the limit of cMD methods in exploring large conformational spaces of such flexible proteins. At the same time, aMD opens to the identification of other descriptors that allows a thorough investigation of the conformational behavior. Starting from these results, since the scope of aMD simulations was to identify minimum energy structures, the following analyses were focused on the frames and the corresponding conformations included in the identified energy minimum.

#### Analysis of descriptors in the minimum of θ angles

Among the several descriptors that we used to analyze the behavior of simulated mAbs, the ϕ angles were computed to describe the rotational movements of Fab domains. Δϕ was computed to determine the shift of each Fab with respect to its starting position, as formalized in Eq. (1).

1 $$\varDelta \phi ={\phi }_{i}-{\phi }_{0}$$

where i is the i-th frame of the trajectory and 0 is the starting frame.

**Fig. 3:**
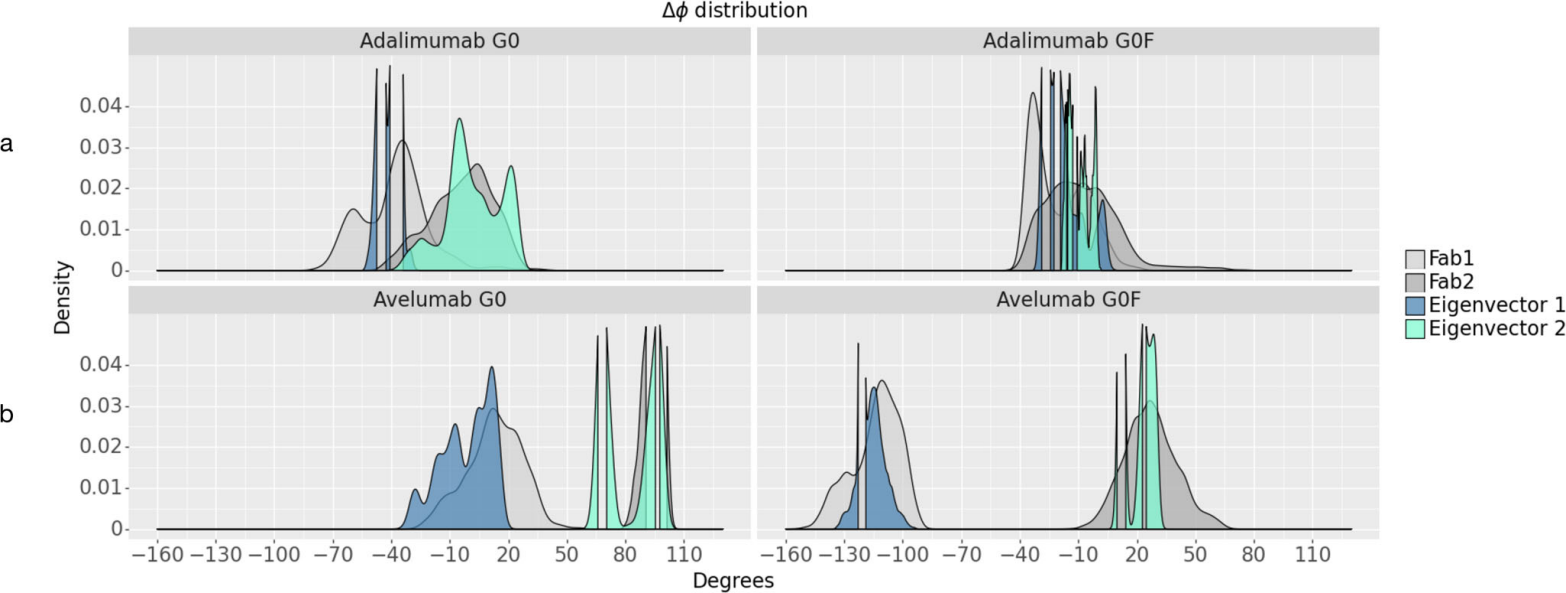
Δϕ distribution along the minimum energy frames and along the first two eigenvectors. Fab1 and Fab2 Δϕ distribution in *κ* (**a**) and *λ* (**b**) mAbs computed for the frames in the energy minimum and along the direction of the first two eigenvectors isolated from the entire trajectories. Density plots show how in each antibody there is at least one eigenvector that can explain the rotations of Fab domains.

Accordingly (Fig. 3), while in the *κ* antibodies there is only a small displacement of Fab domains, *λ* Fab show a higher susceptibility to rotate (at least 100° of shift) even if asymmetrically. An essential dynamics was performed on the overall trajectories to explain the different rotational propensity of the two isotypes. This analysis showed that adalimumab and avelumab are affected by different principal motions and, by computing the Δϕ along the direction of the first two eigenvectors, the distribution of this value is comparable to that computed on the whole trajectory. This suggests that the different rotational propensity is an intrinsic property of the two isotypes. In Supplementary Fig. 6 the normalized PC plot is reported, showing the statistical significance of considering the first two eigenvectors.

**Fig. 4:**
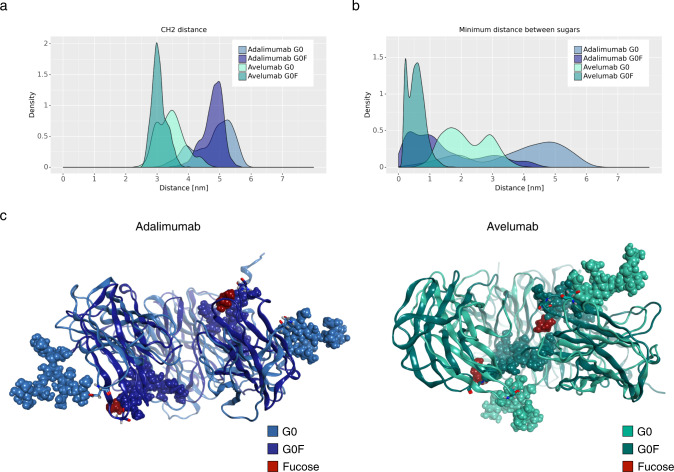
Distribution of CH2 domains distance and of the minimum distance between glycans. Density plot of CH2 distance distribution (**a**) and of the minimum distance between glycan chains (**b**) for each mAb. **c** Structural superposition of representative Fc structures isolated from medoids, showing the different position of G0 (internal) and G0F glycans (external).

A different behavior was also identified between *κ* and *λ* in the context of Fc descriptors. Computing the distance between CH2 domains (Fig. 4a), as for the cMD simulations, it was observed that while *κ* mAbs prefer an open Fc conformation with CH2 distance values globally ranging between 4 and 6 nm, the *λ* ones prefer a closed Fc structure, with CH2 distances between 2 and 4.5 nm. Moreover, the results show that the Fc domain tends to stay in a more closed conformation in G0F species than in G0, according with that previously reported^20^. This data are aligned with the analysis of minimum distance between carbohydrate chains reported in Fig. 4b, showing that, independently from the LC isotype, the fucosylated glycans stay inside the Fc cavity, promoting the closed Fc conformation. On the other hand, G0 glycans prefer to stay outside the Fc and well distanced. In Fig. 4c, medoid structures of Fc domains are reported to highlight the position of glycans with respect to the Fc cavity.

#### Investigation of LC and fucosylation effects

**Fig. 5:**
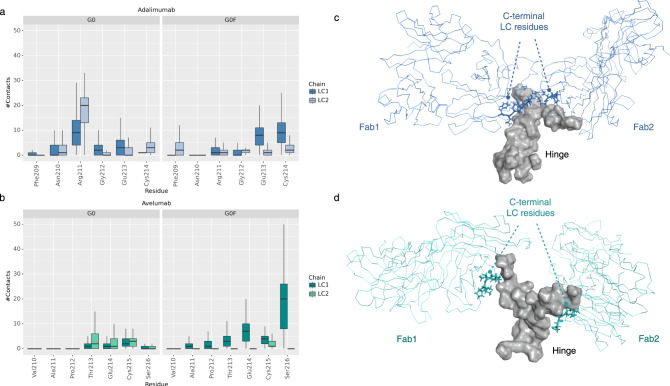
Analysis of the number of contacts between C-terminal LC residues and hinge region. Box plots showing the distribution of the number of contacts between C-terminal LC residues and hinge in adalimumab (**a**) and avelumab (**b**). The number of frames considered in these analyses is: *n* = 2649 for adalimumab G0, *n* = 3440 for adalimumab G0F, *n* = 3170 for avelumab G0, *n* = 4110 for avelumab G0F. Structural representation of G0 adalimumab (**c**) and avelumab (**d**) to highlight the structural proximity between C-terminal LC residues and the hinge. The secondary structure of Fab domains is shown as lines, the LC residues are shown as sticks, and the molecular surface of the hinge is shown in gray. For clarity, Fc is not displayed.

According to the results described above, two effects that modulate the antibody behavior have been observed: the “LC effect”, mainly influencing the Fab rotation and the Fc opening, and the “fucosylation effect”, with a main impact on the Y/T-shaped conformation and the position of glycans inside/outside the Fc cleft. Considering the pivotal role of hinge region in the mAbs flexibility, to investigate the “LC effect”, the contacts between the heavy atoms of the last six (for adalimumab)/seven (for avelumab) C-terminal residues of LC and the hinge were computed, showing a different attitude of the two isotypes in making contacts with the hinge, due to the difference in sequence composition. The results show that G0 adalimumab (Fig. 5a, left panel) makes a higher number of interactions than G0 avelumab (Fig. 5b, left panel), with a key role of Arg211, likely due to the characteristics of substitutions in this region (Supplementary Fig. 1) and to the insertion of Ser216 in *λ* LCs. Considering the G0F antibodies, instead, the effect of fucosylation is translated in an increasing number of contacts for both mAbs, especially in the last 2–3 residues, that for adalimumab include Glu213 and Cys214 (Fig. 5a, right panel), while for avelumab Glu214, Cys215 and Ser216 (Fig. 5b, right panel). On the contrary of G0 species, the combination of LC and fucosylation effects is followed by a strong increase in the number of contacts made by Ser216 in G0F avelumab, suggesting both a susceptibility of this residue to the presence of fucose and a role in modulating the hinge flexibility. In Fig. 5c a schematic representation of the structural proximity between C-terminal LC residues and hinge in G0 antibodies is reported, while in Supplementary Fig. 7 the representation of G0F mAbs is shown. Globally, the lower number of contacts between C-terminal LC and the hinge observed in avelumab could also support the different rotational propensity (Δϕ) of the two antibody species, an aspect that will be further investigated in the future.

**Fig. 6:**
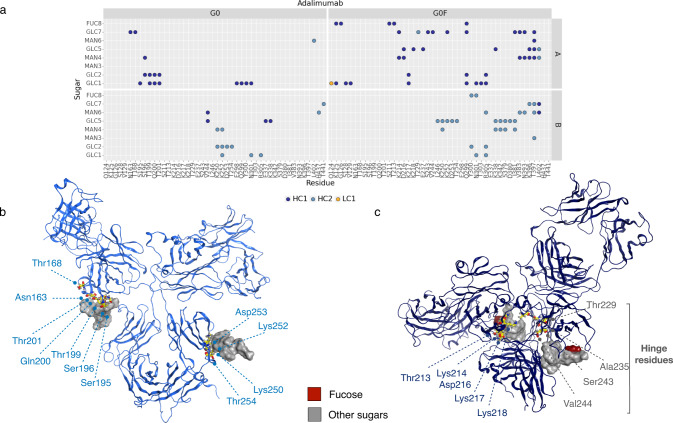
Analysis of H-bonds between glycans and adalimumab. **a** Dot plot showing the specific interactions made by G0 and G0F chains with adalimumab with a specific color code for HC1, HC2, and LC1. Structural representation of the most relevant H-bonds identified between sugars and G0 (**b**) and G0F (**c**) adalimumab. The secondary structure is shown as ribbons, main residues as yellow sticks and the molecular surface of sugars is shown in gray and dark red (fucose).

**Fig. 7:**
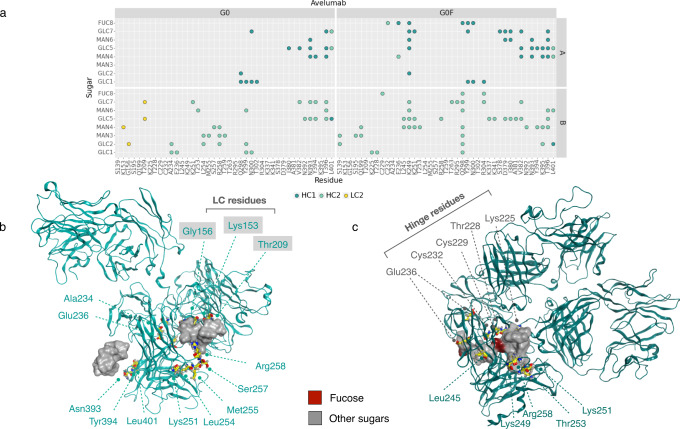
Analysis of H-bonds between glycans and avelumab. **a** Dot plot showing the specific interactions made by G0 and G0F chains with avelumab with a specific color code for HC1, HC2, and LC2. Structural representation of the most relevant H-bonds identified between sugars and G0 (**b**) and G0F (**c**) avelumab. The secondary structure is shown as ribbons, main residues as yellow sticks and the molecular surface of sugars is shown in gray and dark red (fucose).

To evaluate the fucosylation effect, the H-bonds interactions between glycans and the corresponding antibody were computed. A matrix showing the couple of residues involved in these bonds is reported in Figs. 6–7 (panels A). This analysis shows that (i) G0F chains make more contacts than G0 ones; (ii) on the contrary of what observed for heavy atoms interactions, G0 glycans in avelumab make more contacts than in adalimumab and some of these bonds involve LC residues, namely: Lys153, Gly156, and Thr209; (iii) in both G0F mAbs, glycans make interactions directly with the hinge (avelumab) or with near residues (adalimumab) supporting our hypothesis that, modulating the hinge flexibility, the fucose introduces a structural constraint and increases the preference for a T-shaped conformation. In Figs. 6–7 (panels B), a summary of the most relevant interactions between glycans and the antibodies is shown with a particular attention to the residues of LC (G0 avelumab) and the hinge ones. Supplementary Tables 1–4 reports all the found interactions with the associated frequency in the minimum energy frames.

#### Conformational variability of sugars

An analysis of conformational variability of sugar chains was carried out for all the simulated systems considering the minimum energy frames. First, since the use of an enhanced sampling method, such as the aMD, the sampling of the correct φ and ψ dihedral angles identified for each sugars couple was checked. The angles were identified according to the definition reported by Wormald MR and colleagues^24^. Supplementary Figs. 8–10 report the distribution of dihedral values for each sugars couple in G0 and G0F systems compared to values obtained from experimentally solved structures of glycoproteins^24^. Accordingly, in all the systems, the sampling of the dihedrals is consistent with their allowed range. This result validates the use of aMD with CHARMM36 forcefield for the glycans investigated in this work. To further evaluate the position of glycans in Y- and T-shaped conformations, the distance between the center of mass (com) of each glycan chain and itself was computed with respect to the Fc position. The distribution of distance values is shown in Supplementary Fig. 11 and confirmed what already observed with the minimum distance computed between glycan chains A and B in each antibody (Fig. 4b): G0 chains are more flexible than G0F ones and explore a larger conformational space, as pointed out by the higher distance values. Moreover, in avelumab the behavior of the two oligosaccharides is slightly more similar, suggesting a lower flexibility also for G0 chains, that is probably due to the T-shaped conformation assumed by the antibody even in presence of G0 glycans. This analysis was further integrated with a cluster analysis useful to describe the dynamics of sugars. The five most representative glycan structures identified for each chain in each antibody are reported in Supplementary Fig. 12 with the associated cluster population percentage. As a result, all glycans show a certain variability among clusters, suggesting, as expected, a higher flexibility of these molecules with respect to the protein. On the other hand, this analysis confirms that glycans are outside or inside the Fc cavity based on the absence or presence of fucose, respectively, and not based on the antibody shape, Y or T. Moreover, if in adalimumab the behavior of G0 and G0F glycans seems to also influence the different shape of the antibody, in avelumab these aspects are independent. In fact, G0 avelumab assumes a T-shaped conformation even if glycans tend to stay outside from the Fc, thus suggesting, in another way, the role of LC isotype in modulating the antibody dynamics.

## Discussion

Our aim was to investigate via in silico methods the behavior of therapeutic IgG1 with *κ* and *λ* LCs, identifying structural features useful to predict their conformational behavior and bioactivity, and for the first time a minimum energy structure. To the best of our knowledge, this is the first in silico study aimed at comparing these two IgG1 isotypes, both considering the full-length proteins and combining different MD approaches: cMD and aMD simulations. Two representatives of *κ* and *λ* IgG1 LCs, adalimumab and avelumab, in their G0 and G0F forms, were investigated and two effects impacting the flexibility of the mAbs were observed: the “fucosylation effect”, that has been already extensively described^1,11–13,15–17^, and the “LC effect” that we report here for the first time. As already hypothesized in our previous work^20^ and in line with the role of glycosylation in influencing the flexibility of IgG1 demonstrated by Spiteri et al.^21^, the “fucosylation effect” impacts on the Y/T-shaped conformation preference and on the position of glycans inside/outside the Fc. The “LC effect”, instead, was found to modulate the Fab rotation and the Fc opening, as well as contributing to the Y/T-shaped conformation stability, suggesting another player in the conformational features of these biomolecules. Both effects act against the hinge flexibility, being critical for the conformational propensity of the antibody. Based on the results, we can summarize that: (i) considering the G0 forms, while in adalimumab the LC can interact with the hinge modulating its flexibility, in avelumab these interactions are missing due to the differences in the aminoacidic composition. This is translated in a different Fab rotational propensity of the two isotypes (Δϕ angle distribution); (ii) considering the G0F forms, in both antibodies there is an increase of the interaction number both between LC and hinge and between sugars and the protein. G0F sugar chains directly interact with the hinge or near residues, introducing a structural constraint in the molecule. This confirms the already hypothesized effect mediated by fucose on the hinge flexibility; (iii) the effect of fucose is present independently from the LC isotype, but, in G0F avelumab it is enhanced because combined with the effect of *λ* LC, due to the increase in the number of contacts between the C-terminal Cys214, Glu215 and Ser216 and the hinge; (iv) the “LC effect” strongly contributes to the conformational behavior of G0 avelumab, where LC residues are involved in interactions with sugars and not with the hinge as for adalimumab. This supports the hypothesis that the differences in sequence composition of LC could impact the behavior of IgG1, promoting a T-shaped conformation and likely reducing the affinity of λ LC IgG1s for FcγRIIIa. Consequently, our hypothesis is that the impact of fucosylation on ADCC activity of λ LC IgG1s might be lower than *κ* LC IgG1s. Our results about the LC effect, in fact, are strongly in agreement with the experimental study published by Shen et al.^25^. After the deletion of the C-terminal Ser in *λ* LC, that is missing in *κ*, the authors observed not only an increase in stability of the molecule at high pH values, but also an increase in ADCC response, suggesting that the presence of this residue can lead to a compromised IgG1 functionality. On the other hand, the structural role of fucose in modulating the preference of IgG1 for a T-shaped conformation, that we already hypothesized^20^, was further confirmed and explained by the interactions between sugars and the hinge. The implementation of aMD, in fact, allowed us to explore regions of the antibody FES and conformations not observed with classical methods.

The combination of cMD and aMD represents a great enhancement in the deep understanding of the molecular mechanisms that regulate the conformational behavior of this class of molecules. This approach, that has never been used in the context of mAbs, could be further implemented to increase the knowledgebase on the efficacy and the stability of mAbs and to better support the findings on the mechanism of action of this class of biotherapeutics.

This study also lays down the foundations of new approaches for IgG1 dynamics modulation, opening new perspectives in the context of genetic engineering applied to antibody development. Our results, in fact, point out the relevance to carefully consider the LC isotype in the development of novel biotherapeutics. Future in silico protein design approaches and mutagenesis experiments will be useful to validate the role of LC in the antibody flexibility and activity.

## Methods

### Sequence alignment

To identify good templates for our purpose, a sequence alignment among 20 human crystalized κ, 16 human crystalized λ, and 26 marketed fully human IgG1s (21 κ and 5 λ) was performed (Supplementary Note 1 and Supplementary Fig. 1). The human crystalized IgG1 Fab domains were selected by the “Antibody search” tool included in MOE 2020.01^26^, while the marketed molecules were selected from the website “The Antibody Society^27^” and their primary sequences were imported from DrugBank^28^. The “Protein Align” tool by MOE was used to perform the multiple sequence alignment using a BLOSUM62 matrix.

### Homology modeling

The atomistic models of adalimumab and avelumab were built with the same chimeric homology modeling approach by the “Homology model” tool of MOE software^26^. The crystallographic structures of both adalimumab and avelumab Fab domains are available (PDB IDs: 3WD5 and 4NKI, respectively)^29,30^ and were used to model them, while the only solved structure of a whole human IgG1 was used as a template (PDB ID: 1HZH)^31^ to build hinge and Fc portions. The structure of 1HZH that we chose is the one contained in the MOE library of crystalized antibodies, which has been processed by MOE experts with MD simulations. This choice derives from the need to use the same starting conformation for the antibodies to point out differences mediated by glycans. Before performing homology modeling, all the templates were prepared using the “Structure Preparation” tool of MOE, to correct any crystallographic issue, and processed by the “Protonate 3D” tool to assign the ionization states and add missing hydrogens. Both the 3WN5 and 4NKI structures present some missed residues. The last three C-terminal residues in LC of 3WD5 (Gly212, Glu213, and Cys214) were modeled on 1HZH.pdb with the enabled “override template” option. In 4NKI, the first (Gln1) and the last three (Glu214, Cys215 and Ser216) amino acids of the LC were missed. Gln1 was manually added and minimized via the “Protein Builder” tool by MOE, Glu214 and Cys215 were instead modelled on 1HZH.pdb to preserve their orientation with the enabled “override template” option. Ser216 was added once the chimeric model was built and minimized together with the adjacent disulfide bond, in order to preserve the stability of the aglycosylated structure.

The models were then glycosylated. As reported previously^20^, G0 and G0F sugars were attached unit by unit to the conserved Asn297 of adalimumab (Asn301) by the MOE “Carbohydrate builder”. A final minimization step was carried out on entire glycosylated models until the RMS gradient reached 0.01 kcal/mol/Å^2^. For avelumab, G0F glycans, already present in the 1HZH template of the MOE library, were linked to the conserved Asn297 (Asn300) after structural superposition and energy minimized down to an RMS gradient of 0.01 kcal/mol/A^2^. To build the G0 models, the fucose was deleted from the glycan chains on the G0F models, then the glycans and Asn297 were energy minimized again down to an RMS gradient of 0.1 kcal/mol/A^2^.

### Classical and accelerated MD simulations protocol

Three 1 µs cMD simulations were carried out for each model (adalimumab G0 and G0F, avelumab G0 and G0F) using AMBER20^32^ as an integration engine and AMBER10:EHT forcefield^33^, for a total 3 µs of simulated time per each antibody. Two out of three MD simulations of adalimumab had been already performed in our previous work^20^, so they were reanalyzed, and a third replica was computed according to the same protocol. More specifically, systems were configured by MOE and solvated with TIP3P water model and NaCl 0.1 M. The Langevin thermostat was applied for temperature control, the Monte Carlo barostat was used to set constant pressure, and the simulations were carried out in the NPT ensemble (T = 300 K, P = 100 kPa). Sample time was set to 10 ps and the integration time step to 2 fs. Systems were minimized for 5000 steps and a heating phase was performed for 100 ps. Then, an equilibration phase of 100 ps in the NVT and another one of 200 ps in the NPT ensemble were carried out before the production step. Click or tap here to enter text.The XYZ cell dimensions of adalimumab systems are: 186.627 × 160.304 × 90.2284 Å (G0), and 185.041 × 161.404 × 90.1505 Å (G0F); for avelumab: 193.75 × 161.42 × 95.78 Å (G0), and 193.75 × 161.91 × 96.87 Å (G0F).

A short cMD simulation 50 ns long was performed for the four systems with AMBER20^32^ and CHARMM36 forcefield^34^, chosen to investigate the dynamics of sugars more accurately. These simulations were carried out to obtain the average potential energy (EPTOT) and the average dihedral angle energy (DIHED), that were used as input for aMD, as well as a minimized and equilibrated input system to be used for the production phase of aMD. The systems were prepared using the CHARMM-GUI^35^ and solvated with a cubic water box of 181 Å × 3 dimensions with a minimum edge distance of 15 Å and NaCl 0.15 M. Each solvated system was energy minimized for 5000 cycles with a steepest-descent algorithm, applying positional restraints to the protein and sugars and dihedral restraints to sugars. The equilibration phase was carried out for 125 ps in NVT ensemble (T = 300 K) with the Langevin dynamics, the SHAKE algorithm to restrict the vibrations of hydrogen atoms and the particle mesh Ewald (PME)^36^ to calculate electrostatic interactions with a cutoff value of 12 Å. Starting from these equilibrated systems, one aMD simulation 1 µs long was carried out for each antibody. For the aMD production phase the time step was set to 0.002 ps, energy and coordinates were saved every 100 ps, the NPT ensemble was used (T = 300 K; P = 1 bar) with a Langevin dynamics for the temperature control and Berendsen barostat for pressure, and the whole potential was boosted together with an extra boost to the torsions (*iamd* = *3*).

Supplementary Table 5 reports the values of EthreshD, EthreshP, alphaD and alphaP used as input for the simulations and computed as in the following equations^37^:

2 $$EthreshD=DIHED+(4kcal\,mo{l}^{-1}residu{e}^{-1}\ast solute\,residues)$$

3 $$alphaD=(1/5)\ast (4kcal\,mo{l}^{-1}residu{e}^{-1}\ast solute\,residues)$$

4 $$EthreshP=EPTOT+(0.16kcal\,mo{l}^{-1}ato{m}^{-1}\ast number\,of\,atoms)$$

5 $$alphaP=(0.16kcal\,mo{l}^{-1}ato{m}^{-1}\ast number\,of\,atoms)$$

In Supplementary Tables 6 and 7 the setup of systems generated for cMD and aMD simulations, respectively, is reported.

### Analysis of trajectories

RMSD, RMSF and Rg were computed on C-alpha atoms by MDTraj^38^. The analysis of cMD was performed on the concatenated replicas of each system excluding the first 300 ns that were considered as equilibration steps. The movement of Fab domains was described by means of ϕ (longitude) and θ (latitude) angles defined in a reference frame jointed to Fc and centered in the hinge with axes defined as follows: *z* axis collinear to Fc and directed toward Fabs, *x* axis parallel to a vector joining mid-Fc (CH2 regions) and y axis defined by right hand rule. For a more detailed description see the work by Saporiti et al.^20^ An arbitrary threshold of 85° was chosen for θ angle to discriminate between Y- and T-shaped conformations. Specifically, we considered that the only one fully crystalized human IgG1 (PDB ID: 1HZH)^31^, that is classified as a T-shaped conformation^39^, presents θ > 90° for both Fab domains, and we took into account also the conformational variability expected from MD simulations. The distance between the CH2 domains was measured between the glycosylated Asn using MDTraj^38^. Then, box plots were produced to evaluate the statistical significance of the observed values in the total 21,000 frames. For the aMD, a reweighing procedure was applied according to methods described by Miao et al.^40^ using Maclaurin expansion to the 10th order to approximate the free energy surface of the system as a function of θ angles. The RMSD matrices for the cluster analysis (of both cMD and aMD) were generated with CPPTRAJ^41^, while the clusters were obtained using a customized script based on the GROMOS algorithm^42^. In the case of antibodies C-alpha atoms were considered for the analysis, while for glycans the oxygens involved in glycosidic bonds. RMSD-threshold of 7.5 Å and 6.5 Å were used for the antibodies in cMD and aMD, respectively, and the maximum number of clusters was set to 15 and 10, respectively. For glycans clustering the RMSD-threshold was set to 1 Å and the maximum number of clusters to 10. The essential dynamics (ED) was computed on the overall trajectories by the covariance analysis tool of GROMACS 2020.1^20,43^. Then, the resulting trajectories, projected along the first and the second eigenvectors, were filtered by the frames included in the energy minimum that was identified from the FES (computed as function of θ angles) and were used to calculate the Δϕ distribution. The minimum distance between glycan chains was computed by CPPTRAJ^41^ and the “nativecontacts” tool with the “mindist” option, while the distance between the center of mass of each chain and itself was computed with the “distance” tool. For the latter, the trajectories were pre-aligned on the Fc. The contacts between LCs and the hinge region were computed by CPPTRAJ^41^ with the “nativecontacts” tool, considering heavy atoms and a threshold distance of 4 Å. The hydrogen bonds (H-bonds) analysis was computed by a customized python script based on the MDTraj H-bonds identification tool^20^.

### Statistics and reproducibility

Three cMD simulation replicas were performed for each condition. The analyses of cMD trajectories were computed including the last 7000 frames of each replica. Replicas were merged to obtain a total number of frames *n* = 21,000 per each system. The analyses of aMD simulations were performed on the minimum energy frames, i.e., *n* = 2649 for adalimumab G0, *n* = 3440 for adalimumab G0F, *n* = 3170 for avelumab G0, *n* = 4110 for avelumab G0F. For the reproducibility of the analyses, please see the source data behind the plots in Fig. 1(a, b, d) and Fig. 5(a, b) included in Supplementary Data 1. For the reproducibility of the study, please see the input files, the topologies and the starting coordinates of the systems included in Supplementary Data 2.

### Reporting summary

Further information on research design is available in the Nature Portfolio Reporting Summary linked to this article.

## Supplementary information

Supplementary Information

Description of Additional Supplementary Data

Supplementary Data 1

Supplementary Data 2

reporting summary

## Data Availability

All data generated or analyzed during this study are included in this published paper (and its supplementary information files).
