# Supplementary material for: Effect of Fc core fucosylation and light chain isotype on IgG1 flexibility
Source: Commun Biol. 2023 Mar 3;6:237. doi: 10.1038/s42003-023-04622-7 (PMC9982779; doi:10.1038/s42003-023-04622-7)
Supplement: Supplementary file 1 — Supplementary Information [file 42003_2023_4622_MOESM1_ESM.pdf]

SUPPLEMENTARY MATERIAL

Effect of Fc core fucosylation and light chain isotype on IgG1 flexibility

Simona Saporiti[1], Tommaso Laurenzi[1], Uliano Guerrini[1], Crescenzo Coppa[2], Wolf Palinsky[3], Giulia Benigno[1], Luca Palazzolo[1], Omar Ben Mariem[1], Linda Montavoci[1], Mara Rossi[4], Fabio Centola[4§] and Ivano Eberini[5§]

[1]Dipartimento di Scienze Farmacologiche e Biomolecolari, Università degli Studi di Milano, Via Balzaretti 9, 20133 Milan, Italy

[2]Dipartimento di Scienze Farmaceutiche, Università degli Studi di Milano, Sezione di Chimica Generale e Organica "A. Marchesini", Via Venezian, 21 20133 Milano, Italy

[3]Biotech Development Programme, Merck Biopharma, Aubonne, Switzerland (an Affiliate of Merck KGaA, Darmstadt, Germany)

[4]Global Analytical Pharmaceutical Science and Innovation, Merck Serono S.p.A., Rome, Italy (an Affiliate of Merck KGaA, Darmstadt, Germany)

[5]Dipartimento di Scienze Farmacologiche e Biomolecolari & DSRC, Università degli Studi di Milano, Via Balzaretti 9, 20133 Milan, Italy.

§ These authors contributed equally
* Corresponding author; e-mail: fabio.centola@merckgroup.com

Supplementary Note 1: "Case study identification and homology modeling"

To identify good case studies for this investigation, a multiple sequence alignment of 21 λ and 41 κ mAb sequences was performed and the resulting percentage-identity matrix is reported in Supplementary Figure 1. The matrix shows how sequences are highly conserved within the same isotype (> 70% of identity) and how they are less conserved with respect to each other (< 50% of identity), suggesting an inter-class variability. Moreover, the least conserved regions between the two isotypes were identified, showing that, apart from the expected variability in CDRs, together with smaller regions in the variable domains, notable amino acidic differences between the κ and λ LCs can be observed in the constant region of the LCs, such as residues 113-115, 157-172 and 187-193. According to this alignment, adalimumab and avelumab commercial mAbs were identified as well representative of κ and λ isotypes, respectively. Moreover, as a preliminary approach, we decided to consider the variable region of LC not critical for regulating the flexibility of IgG1s, but a comparison between antibodies belonging to the same isotype will be performed in the next future to evaluate also this aspect.

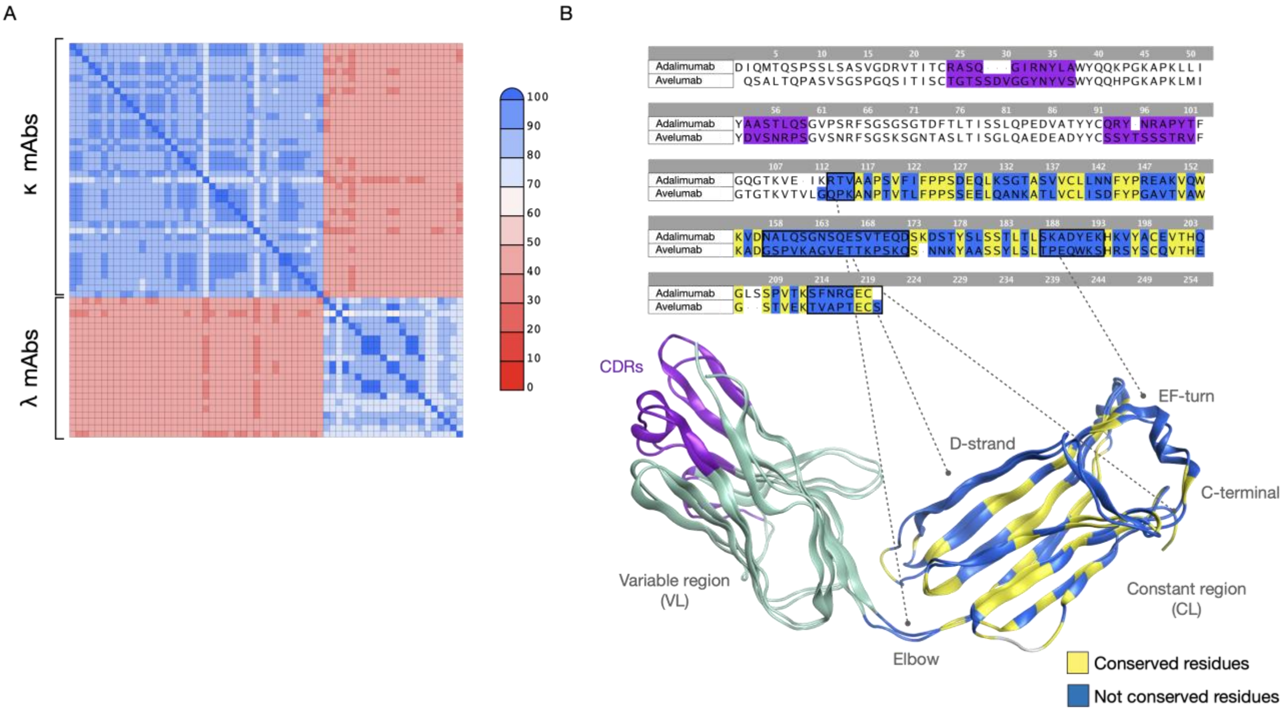

Supplementary Figure 1: Percentage identity matrix and sequence alignment of adalimumab and avelumab. (A) All- against - all percentage identity matrix of λ and κ LCs showing the high identity (> 70%) within the same class and the low identity with respect to the other one (< 50%). (B) On the top, the sequence alignment between adalimumab and avelumab LCs shows a certain variability in the constant domain. The largest different regions are highlighted by black squares; on the bottom, the structural superposition between the two LCs with the largest different regions indicated by arrows. The structure is shown as ribbons colored according to Kabat convention (VL) and sequence identity (CL).

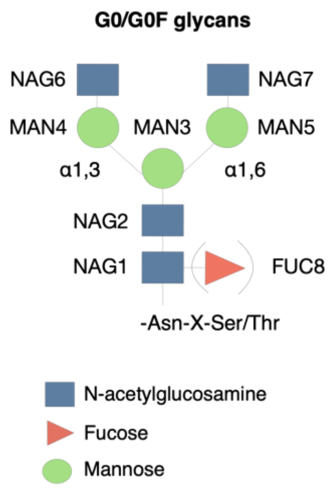

Supplementary Figure 2: SNFG representation of glycans used in this study. Schematic representation according to the Symbol Nomenclature For Glycans (SNFG) scheme of G0/G0F glycans investigated in this study.

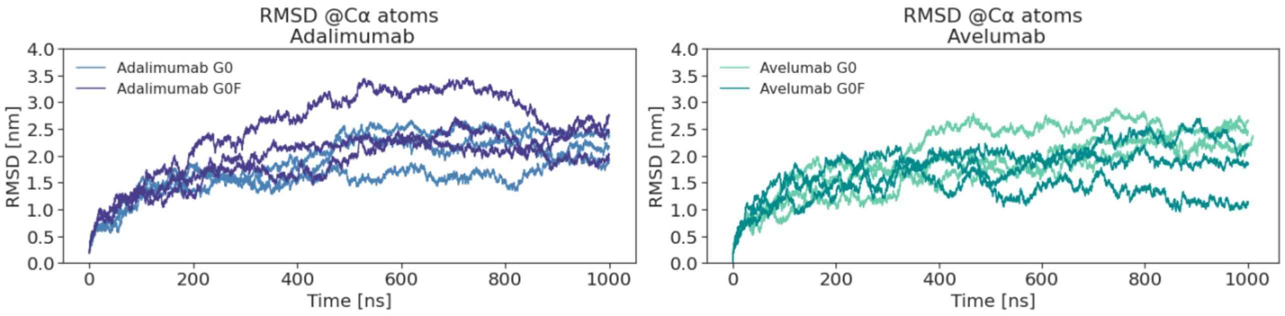

Supplementary Figure 3: RMSD of C-alpha positions in all the cMD systems. RMSD of C-alpha atoms in G0 and G0F adalimumab (left panel) and avelumab (right panel). All the systems globally reach a RMSD plateau after 300 ns of classical MD simulation.

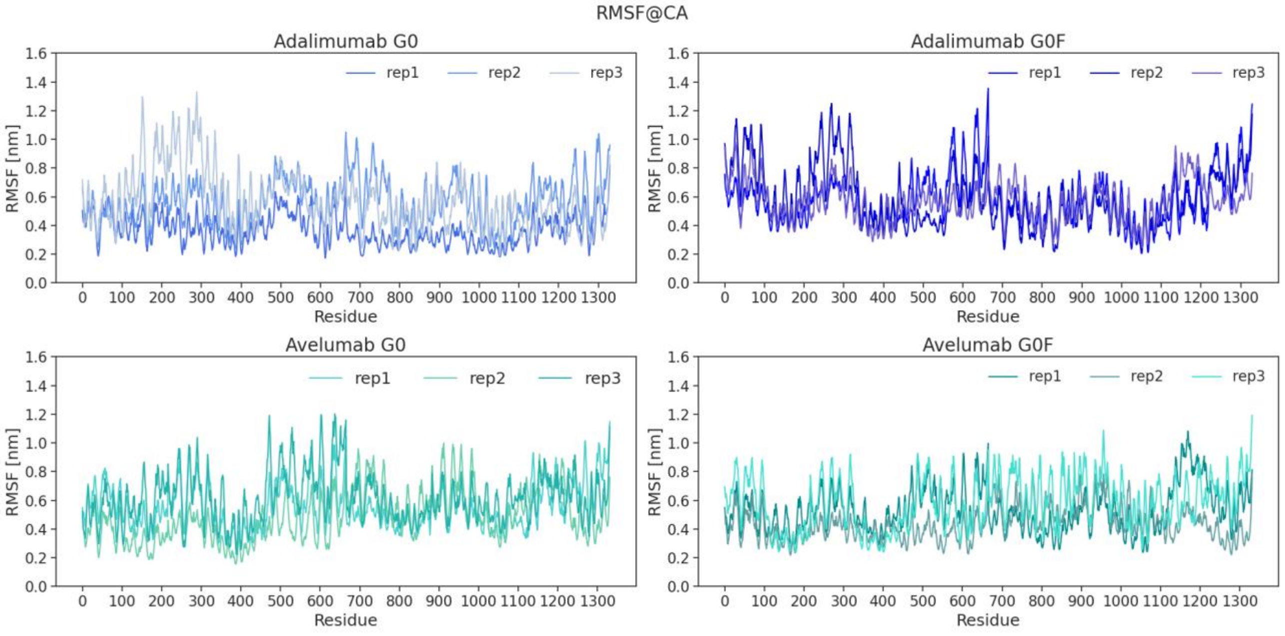

Supplementary Figure 4: RMSF of C-alpha atoms in all the cMD systems. RMSF of C-alpha atoms in G0 and G0F adalimumab (left panel) and avelumab (right panel). Residues are numbered consecutively. Adalimumab: res. 1-214 LC1, res. 215-665 HC1, res. 666-879 LC2, res. 880-1330 HC2; avelumab: res. 1-216 LC1, res. 217-666 HC1, res. 667-882 LC2, res. 883-1332 HC2.

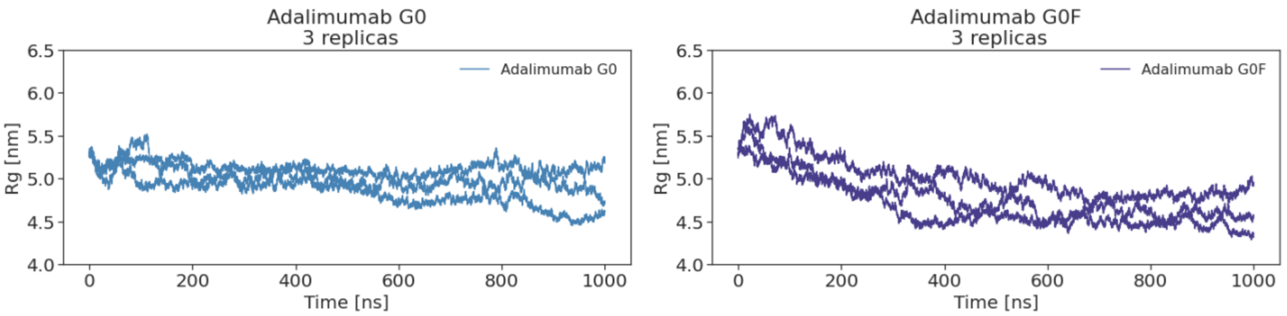

Supplementary Figure 5: Radius of gyration of C-alpha positions in all the cMD systems. Rg of C-alpha atoms in G0 and G0F adalimumab (left panel) and avelumab (right panel). All the systems globally reach a Rg plateau after 300 ns of classical MD simulation.

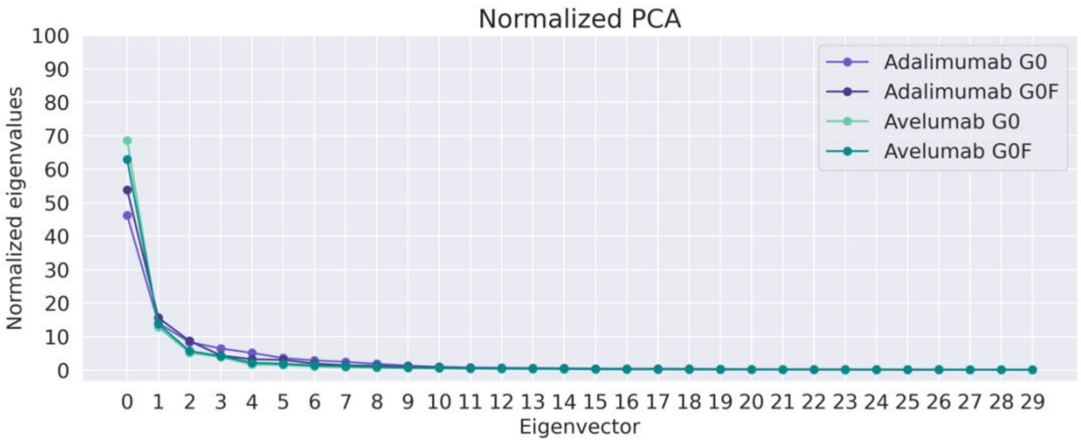

Supplementary Figure 6: Normalized principal component analysis. In the plot the normalized eigenvalues vs eigenvector numbers are reported. The sum of the first two components describes for each antibody more than 60% of the total motions representing the main contributors to the behavior of the molecules.

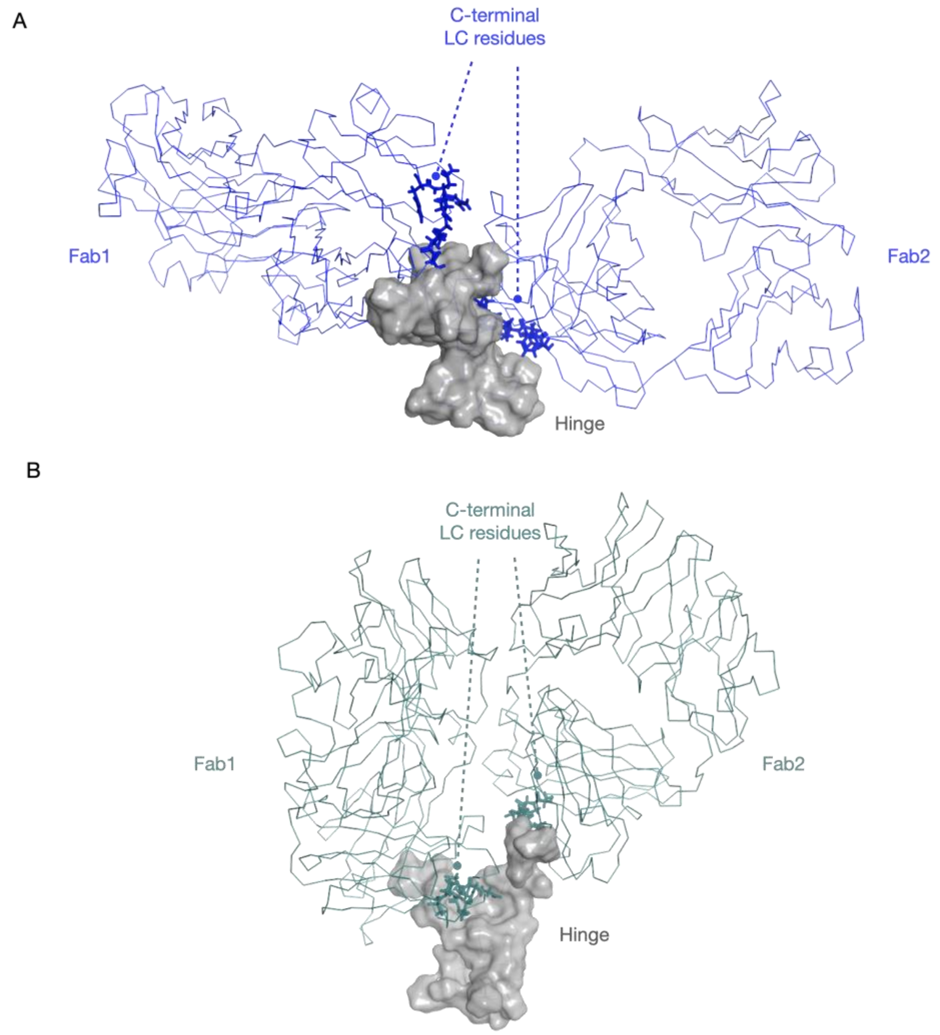

Supplementary Figure 7: Schematic representation of the structural proximity between LC and hinge residues. Structural representation of G0F adalimumab (C) and avelumab (D) to highlight the structural proximity between C-terminal LC residues and the hinge. The secondary structure of Fab domains is shown as lines, the LC residues are shown as sticks and the molecular surface of the hinge is shown in grey. For clarity, Fc is not displayed.

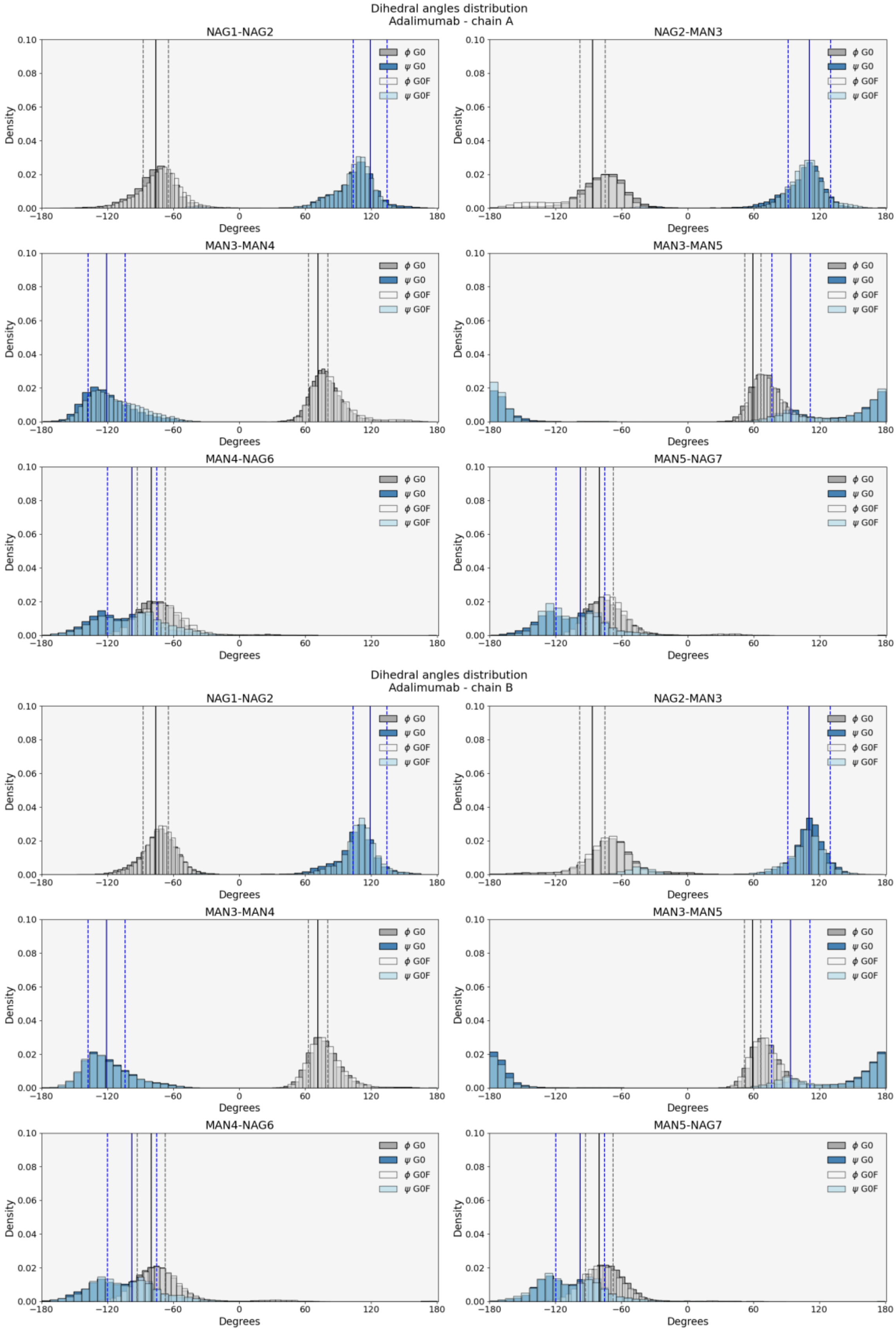

Supplementary Figure 8: Dihedral angles distribution of G0 and G0F chains in adalimumab in the whole aMD trajectories. The distribution of φ and ψ angles of the glycosidic bonds between sugar couples in aMD trajectories of G0 and G0F adalimumab. Lines represent the experimentally calculated mean value of each angle (continuous) ± the standard deviation (dashed lines) as reported by Wormald MR, et al.; Chem Rev. 2002 Feb;102(2):371-86. doi: 10.1021/cr990368i. PMID: 11841247.

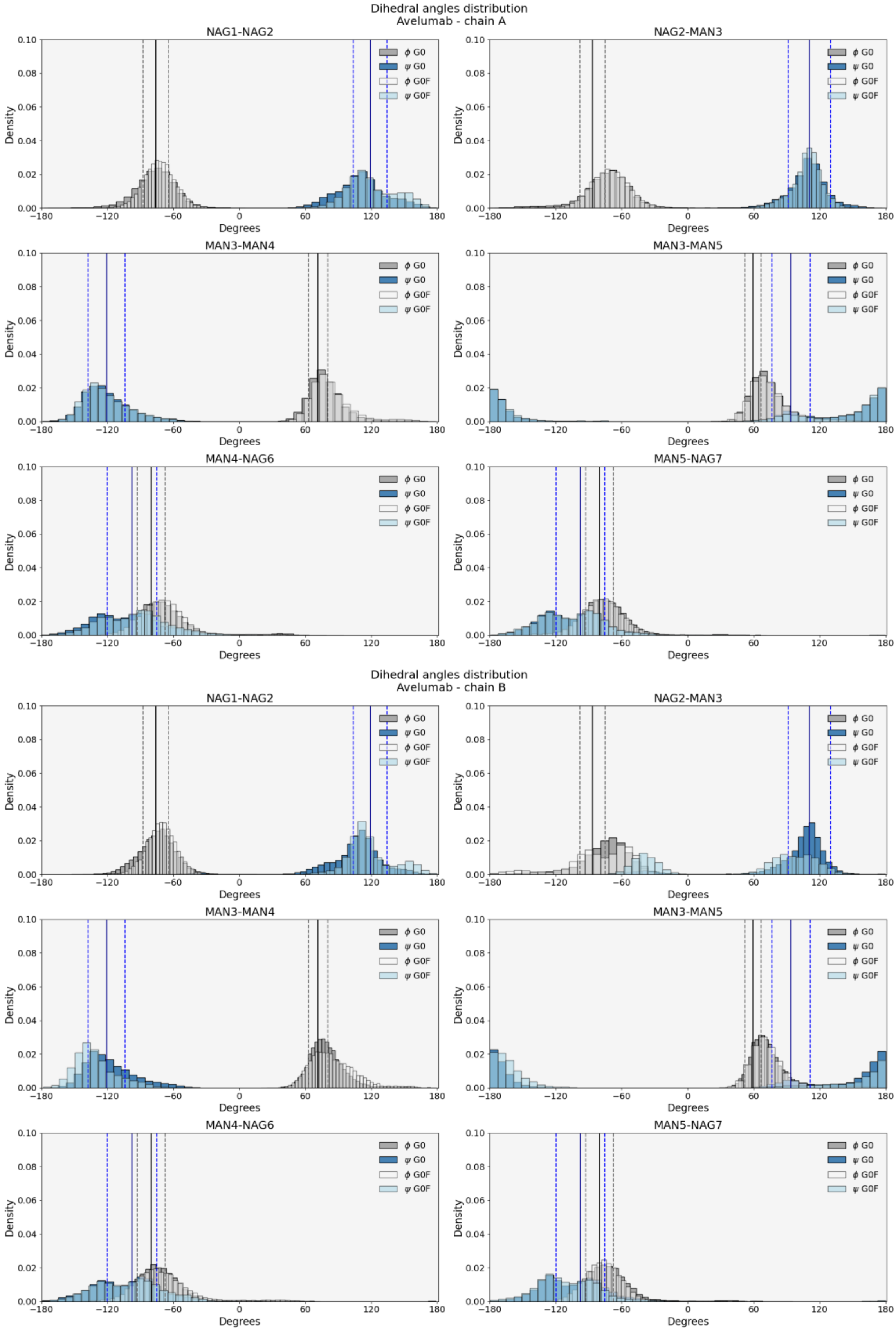

Supplementary Figure 9: Dihedral angles distribution of G0 and G0F chains in avelumab in the whole aMD trajectories. The distribution of ϕ and ψ angles of the glycosidic bonds between sugar couples in aMD trajectories of G0 and G0F avelumab. Lines represent the experimentally calculated mean value of each angle (continuous) ± the standard deviation (dashed lines) as reported by Wormald MR, et al.; Chem Rev. 2002 Feb;102(2):371-86. doi: 10.1021/cr990368i. PMID: 11841247.

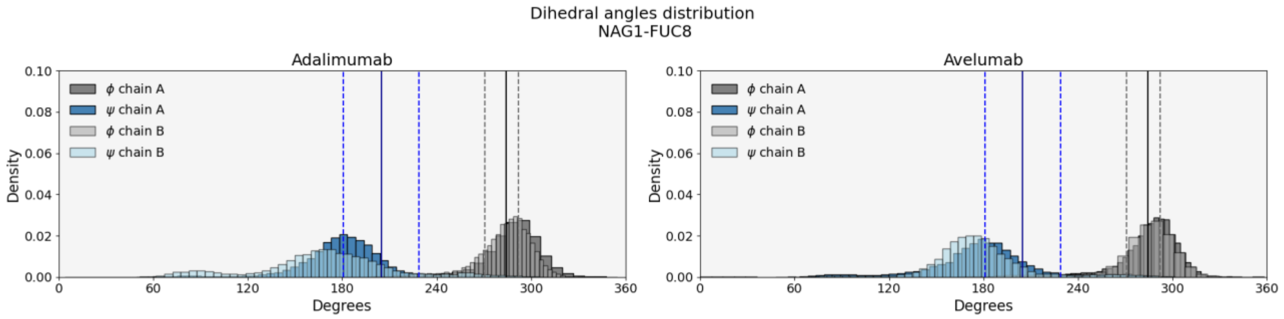

Supplementary Figure 10: Dihedral angles distribution of NAG1-FUC8 couple in G0F chains in the whole aMD trajectories. The distribution of ϕ and ψ angles of the NAG1-FUC8 glycosidic bond in adalimumab (on the left) and avelumab (on the right). Lines represent the experimentally calculated mean value of each angle (continuous) ± the standard deviation (dashed lines) as reported by Wormald MR, et al.; Chem Rev. 2002 Feb;102(2):371-86. doi: 10.1021/cr990368i. PMID: 11841247.

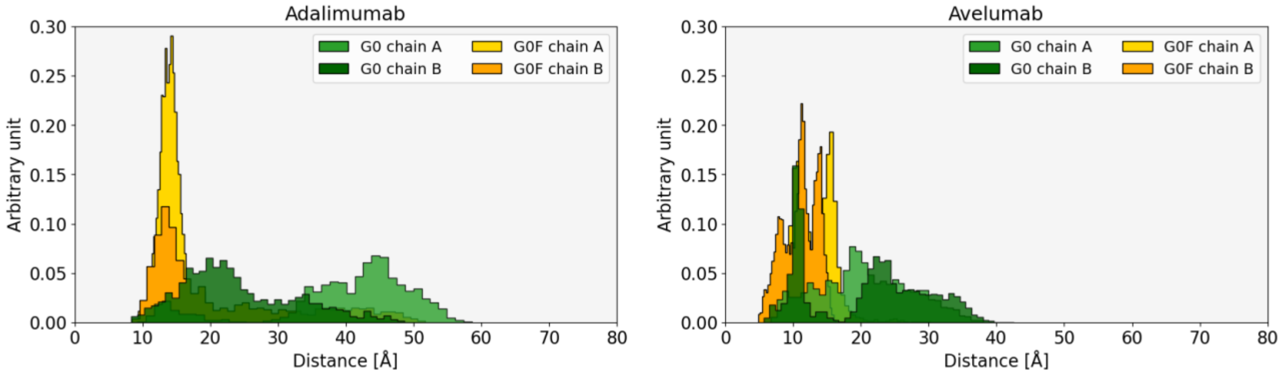

Supplementary Figure 11: Distance distribution of glycan chains center of mass from itself. The distribution of the distance between the center of mass of each glycan chain from itself and computed with respect to the Fc position in the minimum energy frames. Plots show that in both antibodies G0F chains are less flexible than G0 ones and that in particular in avelumab a lower flexibility of G0 chains is observed than in G0 adalimumab.

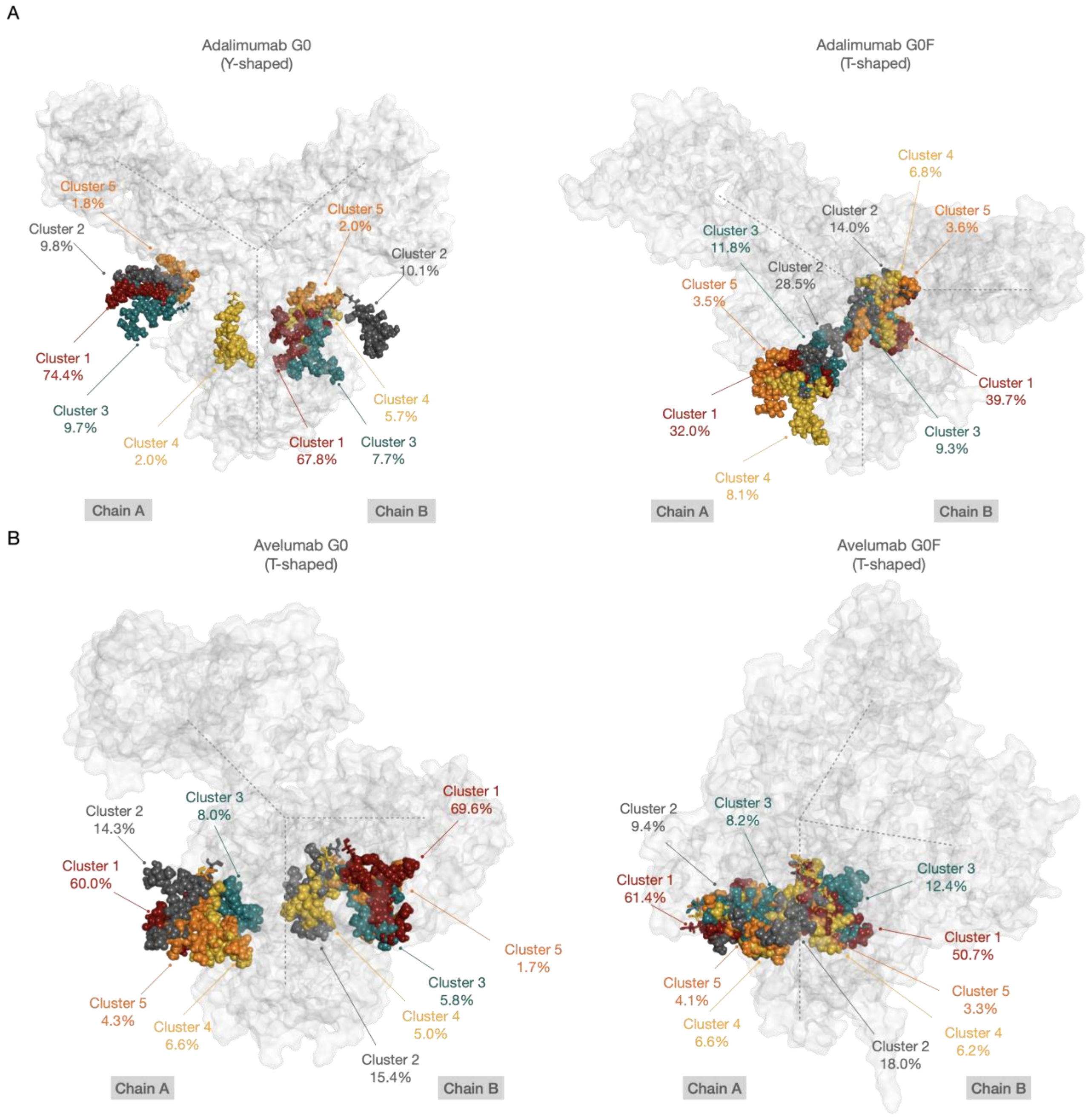

Supplementary Figure 12: Cluster analysis of glycan chains in all antibodies. (A) Five medoid structures isolated from the most representative clusters of G0 (left) and G0F (right) adalimumab glycan chains with the associated cluster population percentage. (B) Five medoid structures isolated from the most representative clusters of G0 (left) and G0F (right) avelumab glycan chains with the associated cluster population percentage. Antibodies are represented as grey molecular surface; dashed lines remark the Y- or T-shaped conformation found in each antibody; sugars and the glycosylated Asn are shown as spheres and sticks colored by cluster, respectively.

Supplementary Table 1: H-bonds interactions between G0 adalimumab and G0 sugar chains with the associated frequency in the selected energy minimum frames. In green and bold, those interactions that occur with a frequency up to 10%.

Residue	Chain	Sugar	Sugar chain	Frequency (%)
N163	HC1	GLC7	A	1.17
T168	HC1	GLC7	A	1.74
S195	HC1	GLC1	A	1.28
S196	HC1	GLC2, MAN4	A	1.17; 1.01
**T199**	HC1	**GLC1,** GLC2	A	**10.04;** 4.98
Q200	HC1	GLC2, GLC1	A	5.25; 4.53
T201	HC1	GLC1, GLC2	A	3.96; 7.70
V244	HC1	MAN6, GLC5	B	1.01; 2.91
K250	HC2	MAN4, GLC2	B	1.02; 1.28
K252	HC2	MAN4, GLC2, GLC1	B	1.17; 2.68; 3.70
D253	HC2	GLC2	B	1.43
T254	HC2	GLC2	B	1.09
E298	HC1	GLC1	A	5.51
Q299	HC1	GLC1	A	4.53
Y300	HC1	GLC1	A	6.26
**N301**	HC1, HC2	**GLC1**	A/B	**25.56; 31.14**
R305	HC2	GLC1	B	4.11
E337	HC1	GLC5	B	3.13
K338	HC1	GLC5	B	1.96
L402	HC2	MAN6	A	1.47
H437	HC2	MAN6	B	2.00
T441	HC2	GLC7	B	1.17

Supplementary Table 2: H-bonds interactions between G0F adalimumab and G0F sugar chains with the associated frequency in the selected energy minimum frames. In green and bold, those interactions that occur with a frequency up to 10%.

Residue	Chain	Sugar	Sugar chain	Frequency (%)
Q124	LC1	GLC1	A	2.50
K125	HC1	GLC1, FUC8	A	7.62; 7.88
**G126**	HC1	**FUC8**	A	**12.88**
S128	HC1	GLC1	A	2.18
V129	HC1	GLC1	A	7.91
S211	HC1	FUC8	A	1.02
**T213**	HC1	**FUC8**	A	**10.93**
K214	HC1	MAN4, GLC7	A	4.68; 2.50
D216	HC1	MAN4, GLC5	A	8.17; 1.19
K217	HC1	GLC1, GLC2	A	3.46; 2.53
K218	HC1	GLC5	A	7.82
T229	HC2	GLC7	A	1.02
E237	HC1	GLC5	A	1.08
S243	HC1	GLC7	A	23.43
V244	HC1	GLC7	A	6.74
L246	HC2	GLC5	B	1.22
K250	HC2	MAN4, GLC5	B	1.28; 1.63
K252	HC2	GLC5, GLC7	B	1.10; 1.80
D253	HC2	GLC5	B	1.74
T254	HC2	GLC5	B	3.02
**Q299**	HC1	GLC1, **GLC2,** GLC7, FUC8	A	7.79; **10.73;** 5.93; 2.03
Y300	HC2	GLC2, FUC8	B	3.75; 5.15
**N301**	HC1, HC2	**GLC1, FUC8**	A/B, B	**18.28; 14.36;** 1.60
T303	HC1	GLC1	A	5.20
R305	HC1	GLC1, GLC2	A	2.79; 14.62
R305	HC2	GLC1, GLC2, MAN6	B	3.75; 1.02; 2.18
K338	HC1, HC2	MAN4, GLC5	B, A/B	2.38; 1.13; 3.55
K342	HC2	MAN4, GLC5	B	1.57; 3.28
**S379**	HC2	MAN4, **GLC5**	B	2.35; **17.79**
D380	HC2	GLC5	B	1.40
I381	HC1, HC2	MAN4, GLC5, GLC7	B, B, A	1.45; 1.95; 5.67
**V383**	HC1, HC2	MAN4, **MAN6,** GLC7	A, B, A	2.06; **12.73;** 19.71
N393	HC2	MAN4, MAN6, GLC7	A, B, A,	1.16; 2.53; 1.45
K396	HC1, HC2	MAN4, GLC5, GLC7	A, A, B	9.91; 2.21; 2.79
**T397**	HC1	MAN4, MAN6, **GLC5,** GLC7	A	2.38; 4.94; **10.38;** 9.62
**T397**	HC2	MAN3, **MAN6, GLC7**	B	1.08; **12.24; 13.60**
L402	HC1	MAN6, GLC7	B	1.40; 2.44
L402	HC2	**MAN4, GLC5**	A	**32.85; 13.69**

Supplementary Table 3: H-bonds interactions between G0 avelumab and G0 sugar chains with the associated frequency in the selected energy minimum frames. In green and bold, those interactions that occur with a frequency up to 10%.

Residue	Chain	Sugar	Sugar chain	Frequency (%)
K153	LC2	MAN4	B	3.72
G156	LC2	GLC2	B	1.64
T209	LC2	GLC5, GLC7	B	1.20; 3.94
A234	HC2	GLC1, GLC2	B	2.15; 1.14
E236	HC2	GLC1	B	2.97
K251	HC2	GLC7	B	4.13
T253	HC2	MAN6	B	2.59
L254	HC2	GLC2, MAN3	B	7.57; 5.49
M255	HC2	MAN3	B	7.54
S257	HC2	MAN4	B	4.29
R258	HC2	GLC2, MAN3, MAN4	B	1.64; 1.14; 4.42
T259	HC2	MAN3	B	1.55
Q298	HC1	GLC1, GLC2	A	5.33; 3.53
**Y299**	HC1, HC2	**GLC1,** GLC2, MAN6	A/B, B, B	1.10; **11.99;** 7.35; 1.14
**N300**	HC1, HC2	**GLC1, GLC7**	A/B, A	**11.51; 19.62;** 1.39
T302	HC1	GLC1	A	1.42
I380	HC1	GLC5	A	1.61
V382	HC1, HC2	GLC5, GLC7	A, B	1.07; 21.42
N392	HC2	GLC5	B	1.04
N393	HC1, HC2	GLC5, MAN4, MAN6, GLC7	A/B, A, A, B	1.07; 2.43; 1.55; 1.10; 1.55
Y394	HC1, HC2	MAN4, GLC5, GLC7	A, B, B	1.20; 2.30; 1.23
T396	HC2	GLC5, MAN6, GLC7	B	2.43; 3.31; 7.03
T396	HC1	MAN4, GLC5, GLC7	A	1.83; 1.77; 3.41
L401	HC1, HC2	GLC5, GLC7	A/B, A	1.07; 6.91; 1.89

Supplementary Table 4: H-bonds interactions between G0F avelumab and G0F sugar chains with the associated frequency in the selected energy minimum frames. In green and bold, those interactions that occur with a frequency up to 10%.

Residue	Chain	Sugar	Sugar chain	Frequency
S139	HC2	GLC2, MAN3	B	2.14; 3.77
S195	HC2	MAN3	B	1.34
Q199	HC2	MAN3, MAN4	B	1.09; 4.04
K225	HC2	GLC1	B	12.14
T228	HC2	GLC1	B	8.30
C229	HC2	FUC8	B	1.41
C232	HC2	FUC8	A	3.75
E236	HC1, HC2	MAN4, FUC8	A	1.12; 4.48
L245	HC2	MAN4, GLC5	B	1.95; 4.99
**K249**	HC1	GLC2, GLC5, MAN6, **GLC7,** FUC8	A	1.31; 1.09; 3.48; **19.39;** 7.66
K249	HC2	MAN4, GLC5, MAN6, GLC7	B	1.85; 2.68; 4.84; 4.48
K251	HC1, HC2	MAN4, GLC5, GLC7	B, B, A	1.27; 9.71; 1.41
T253	HC2	MAN4	B	2.00
R258	HC2	GLC5	B	1.65
T263	HC2	GLC7	B	2.02
R295	HC2	GLC7	B	6.30
Q298	HC1	FUC8	A	5.01
**Q298**	HC2	GLC1, **GLC2,** MAN3, MAN4, **MAN6,** GLC7, FUC8	B	7.86; **12.12;** 7.76; 5.18; **15.94;** 7.03; 6.20
Y299	HC1	GLC1, GLC7, FUC8	A	2.92; 1.36; 14.79
**N300**	HC1	**GLC1,** FUC8	A	**10.83;** 5.28
**R304**	HC1, HC2	GLC1, **GLC7,** FUC8	A, B, B	6.79; **24.36;** 3.84
K337	HC2	GLC5	B	2.19
K341	HC2	GLC5	B	1.56
S378	HC1	GLC7	A	3.99
**D379**	HC1, HC2	**GLC5,** MAN6, **GLC7**	B, A, A	**11.75;** 2.85; **18.15**
**I380**	HC1, HC2	**GLC5,** MAN6, **GLC7**	B, A, A	**11.73;** 1.68; **13.99**
**A381**	HC2	**GLC5**	B	**19.29**
**V382**	HC2	MAN4, **GLC5, GLC7**	A, A/B, A/B	3.72; **24.74; 30.90; 13.14; 11.90**
**N392**	HC2	**MAN4**	B	**10.19**
N393	HC1	MAN4, MAN6, GLC5	A	5.18; 2.82; 8.10
Y394	HC1, HC2	MAN4, GLC5	B, A	4.57; 8.52
K395	HC1	GLC2, MAN4, GLC7	B, A/B, A	3.28; 1.58; 1.27; 1.07
**T396**	HC2	**MAN4, MAN6,** GLC5	B	**41.05; 19.66;** 2.26
**T396**	HC1	MAN4, MAN6, **GLC5,** GLC7	A	2.85; 6.08; **11.63;** 1.95
**L401**	HC1, HC2	**GLC2,** MAN4, **GLC5,** MAN6	B, A, A, B	**46.91;** 1.70; **17.10;** 1.73

Supplementary Table 5: Accelerated molecular dynamics parameters in kcal/mol.

	Adalimumab G0	Adalimumab G0F	Avelumab G0	Avelumab G0F
EthreshD	18,730	18,805	18,498	18,590
EthreshP	-1,750,000	-1,750,000	-1,747,000	1,774,000
alphaD	1,075	1,076	1,077	1,078
alphaP	90,000	90,000	90,000	91,424

Supplementary Table 6: Systems setup of cMD simulations with AMBER10:EHT forcefield.

System	Box dimensions (XYZ)	# Atoms	# Solvent molecules	Salt concentration
Adalimumab G0	186.63 × 160.30 × 90.23 Å	315,103	294,510	0.1 M
Adalimumab G0F	185.04 × 161.40 × 90.15 Å	314,858	294,225	0.1 M
Avelumab G0	193.75 x 161.42 x 95.78 Å	298,065	277,687	0.1 M
Avelumab G0F	193.75 x 161.91 x 96.87 Å	302,842	282,423	0.1 M

Supplementary Table 7: Systems setup of cMD simulations with CHARMM36 forcefield and aMD simulations.

System	Box dimensions (XYZ)	# Atoms	# Solvent molecules	Salt concentration
Adalimumab G0	181 Å x 3	562,875	541,934	0.15 M
Adalimumab G0F	181 Å x 3	562,981	542,000	0.15 M
Avelumab G0	181 Å x 3	563,125	542,390	0.15 M
Avelumab G0F	181 Å x 3	571,400	550,619	0.15 M
