# Supplementary material for: Effect of Fc core fucosylation and light chain isotype on IgG1 flexibility
Source: Commun Biol. 2023 Mar 3;6:237. doi: 10.1038/s42003-023-04622-7 (PMC9982779; doi:10.1038/s42003-023-04622-7)
Supplement: Supplementary file 2 — Description of Additional Supplementary Data [file 42003_2023_4622_MOESM2_ESM.docx]

**Description of Additional Supplementary Files**

**File name:** Supplementary Data 1

**Description:** The source data behind the graphs in Figures 1 (a,b,d) and 5 (a,b).

**File name:** Supplementary Data 2

**Description:** Coordinates, topology and input files of molecular dynamics simulations.
